# A double-edged effect of hypoxia on astrocyte-derived exosome releases

**DOI:** 10.3389/ebm.2025.10559

**Published:** 2025-05-21

**Authors:** Yang Jie Tseng, Hui-Ju Huang, Chien-Hui Lin, Anya Maan-Yuh Lin

**Affiliations:** ^1^ Ph.D. Program in Regulatory Science and Policy, National Yang-Ming Chiao-Tung University, Hsin-Chu, Taiwan; ^2^ Department of Medical Research, Taipei Veterans General Hospital, Taipei, Taiwan; ^3^ Institute of Physiology, National Yang-Ming Chiao-Tung University, Hsin-Chu, Taiwan; ^4^ Department of Pharmacy, National Yang-Ming Chiao-Tung University, Hsin-Chu, Taiwan

**Keywords:** hypoxic preconditioning, double-edged role, exosomes, hemin, CTX-TNA2

## Abstract

Exosomes are the smallest extracellular vesicles secreted from cells, carrying different cargos, including nucleic acids, proteins and others which transfer from cells to cells. The properties of exosomes depend on the donor cells. Hypoxia, referring to a sublethal and insufficient oxygen supply, reportedly influences exosome secretion of hypoxic cells. In the present study, we focused on the effects of hypoxia on exosomes obtained from CTX-TNA2 astrocyte cells exposed to different durations of hypoxia followed by normoxia as a model of hypoxic preconditioning. To evaluate the functions of exosomes, primary cultured cortical neurons were treated with hemin, a potent neurotoxin. Our sulforhodamine B assay showed that incubation of hemin (30 μM) consistently induced neuronal death. Co-incubation of exosomes from CTX-TNA2 cells subjected to 2 hr-hypoxia plus 6 hr-renormoxia (2H/6R exosomes), but not 12 hr-hypoxia plus 24 hr-renormoxia (12H/24R exosomes), attenuated hemin-induced cell death and reduction in growth associated protein 43 level (a biomarker of neurite outgrowth). Western blot assay demonstrated that 2H/6R exosomes attenuated hemin-induced elevations in inducible nitric oxide synthase (iNOS) and cyclooxygenase-2 (COX-2) levels (two proinflammatory biomarkers) as well as heme oxygenase-1 (HO-1). In contrast, 12H/24R exosomes did not alter hemin-induced elevation in HO-1 but further augmented hemin-induced increases in iNOS and COX-2. Moreover, 2H/6R exosomes attenuated hemin-induced reduction in glutathione hydroperoxidase 4 (a biomarker of ferroptosis) and elevation in active caspase 3 (a biomarker of apoptosis) while 12H/24R exosomes did not effectively alter hemin-induced programed cell death. In conclusion, our study showed that 2H/6R exosomes possessed neuroprotective activities while 12H/24R exosomes had mild pro-inflammatory activities, suggesting that different hypoxic preconditionings influenced CTX-TNA2 cells which then secreted exosomes with differential biological activities. These findings highlight a double-edged role of hypoxia on exosome functions.

## Impact statement

To delineate the role of hypoxic preconditioning on the exosome’s secretion and functions, the CTX-TNA2 cells were exposed to different durations of hypoxia plus renormoxia and were found to secrete exosomes with opposite functions. One was to attenuate hemin-induced neurotoxicity and the other was to augment hemin-induced neuroinflammation. Our data suggests that in addition to exosomes secretion, hypoxic treatment plays a crucial role in modulating exosomes functions.

## Introduction

Cells are known to secrete small membrane-bound vesicles, namely, extracellular vesicles (EVs). Extracellular vesicles differ in size, density, morphology, contents, and biomarkers [1–3]. Exosomes are the smallest subgroup of EVs, ranging from 30 to 160 nm. Exosomes carry various biomolecules, including proteins, lipids, and nucleic acids (RNA, DNA) which are reportedly transferred from donor cells to target cells [4]. Accordingly, the function of exosomes depends on the status of donor cells [2]. Protective exosomes from good cells are suggested to carry “good” cargos while detrimental exosomes from bad cells carry “bad” cargos [4, 5]. Many non-clinical and clinical studies have demonstrated cytoprotective roles of exosomes against various diseases [1, 5–10]. For example, non-clinical studies showed that exosomes from cardiac fibroblasts inhibited heart failure and myocardial ischemic damage [7]. Furthermore, exosomes derived from umbilical cord mesenchymal stem cells (MSC) reportedly prevented acute liver damage in mice [8]. Exosomes from hypoxic MSC exert its protective action via attenuating neurological deficits, and program cell death in stroke and Alzheimer’s models [9] as well as promoting bone fracture healing [10]. At the same time, many clinical trials focused on the protective effects of exosomes on renal diseases, type 1 diabetes, osteoarthritis, stroke, Alzheimer’s disease, and cancers [1, 6].

Hypoxia refers to a condition in which oxygen supply is insufficient in cells, tissues, organs, and systems [11, 12]. A variety of protective functions via hypoxia-induced adaptation has been demonstrated. For example, a non-lethal hypoxia in the tumor microenvironment is reportedly cytoprotective to cancer cells [13, 14]. Furthermore, our previous studies have demonstrated that hypoxic preconditioning is neuroprotective against kainic acid-induced neurotoxicity [15] and ischemic stroke [16]. The proposed mechanisms underlying hypoxia-induced adaptation included induction of hypoxia-inducible factor-α, a transcription factor which is reportedly up-regulated to activate genes and is responsible for adaptation-related cellular responses [12]. Furthermore, hypoxia-induced adaptation may be mediated via regulation of anti-oxidative defense systems, induction of autophagy [17], exosome secretion [4, 10] and others.

Due to the intersection of hypoxia and exosomes, the present study focused on the effects of hypoxia on CTX-TNA2 cells-derived exosomes using hemin-induced neurotoxicity, an intracerebral hemorrhage (ICH) *in vitro* model [18]. Astrocytes are reportedly involved in homeostasis and defense of the central nervous system (CNS) [19]. During the insults, astrocytes are activated and may damage the nearby cells. However, recent studies proposed a neuroprotective role of astrocytes in the astrocyte polarization [20] following brain injury. These findings indicate that astrocytes may play beneficial or detrimental roles in CNS neurodegenerative diseases [19–21]. Given that astrocytes are the most numerous cell type in the brain, astrocytes-derived exosomes may have a substantial influence on the progression of CNS neurodegenerative disorders, including ICH. In the present study, CTX-TNA2 astrocyte cells were exposed to different durations of hypoxia and subsequent renormoxia, including 2-h hypoxia followed by 6-h normoxia (2H/6R) and 12-h hypoxia followed by 24-h normoxia (12H/24R). Both 2H/6R exosomes and 12H/24R exosomes were collected and their functions were characterized. Our data showed that 2H/6R exosomes attenuated hemin-induced neurotoxicity while 12H/24R exosomes possessed proinflammatory activities, indicating that the role of hypoxia is double-edged in exosomes’ functions.

## Materials and methods

### Chemicals

The chemicals used were hemin (Sigma, St. Louis, MO, United States). Hemin was dissolved in ammonia water and pH value was corrected to pH 7.4. Hemin was diluted with DMEM or Neurobasal medium (NB, Thermo Fisher Scientific, Waltham, MA, United States).

### Hypoxic preconditioning of CTX-TNA2 cells

CTX-TNA2, an astrocyte cell line isolated from the cortex of 1-day old rats, was purchased from Bioresource Collection and Research Center (Taiwan) and maintained in Dulbecco’s Modified Eagle Medium (Sigma) supplemented with 10% (v/v) fetal bovine serum (Cytiva, Marlborough, MA, United States) and 1% penicillin-streptomycin-amphotericin B (Cytiva) in an incubator under 5% CO2 at 37°C. Prior to the hypoxic preconditioning, CTX-TNA2 cells were seeded at a density of 1 × 10^6^ cells in a 10-cm culture dish and were incubated in 5 mL NB without exosomes. Hypoxic treatment was conducted by incubating cells in an incubator with 1% oxygen. Two hypoxic preconditionings were designed as follows. One hypoxic treatment was to expose CTX-TNA2 cells to 2-h hypoxia followed by 6-h normoxia. The other was to expose CTX-TNA2 cells to 12-h hypoxia followed by 24-h normoxia.

### Exosomes preparation

At the end of hypoxia-renormoxia treatments, exosomes obtained from the culture medium were isolated using ExoQuick (System Biosciences, Palo Alto, CA, United States). In brief, the culture medium from 10-cm culture dishes was collected and centrifuged at 2600 × *g* for 30 min at 4°C. The supernatant was transferred to a new centrifuge tube. Five milliliters of culture medium mixed with 1 mL of ExoQuick and the tube was placed in a refrigerator at 4°C overnight. The supernatant was collected and centrifuged at 1,500 × *g* for 30 min at 4°C. Again, the supernatant was collected and centrifuged at 1,500 × *g* for 5 min. After removing the remaining supernatant, the exosome pellet was re-dissolved in PBS at 4°C and stored at 4°C. Approximately, 5 × 10^8^ exosome particles obtained from 1 × 10^6^ cells in a 10-cm culture dish were used for further experiments as 1-fold (1x) of exosomes.

### Nanoparticle tracking analysis and detection of zeta potentials

Nanoparticle Tracking Analysis (NTA) was employed to characterize exosomes, including size distribution and exosome particle concentration by NanoSight NS300 (Malvern Panalytical, Malvern, UK). This technique was capable of detecting vesicles with sizes between 0.05 and 1 μm. At the same time, zeta potential of exosomes was analyzed using the Nano ZS (Malvern Panalytical).

### Transmission electron microscopy

An aliquot of exosomes was applied onto an electron microscopy grid and incubated for 5 min and fixed with 2% paraformaldehyde for 5 min. The samples were counterstained with 1% uranyl acetate for 1 min, air-dried overnight, and observed using a Transmission Electron Microscope JEM-1400Plus (JEOL Ltd., Tokyo, Japan).

### RNA sequencing analysis

MicroRNA(miRNA) libraries were prepared using the QIAseq miRNA UDI Library Kit (Qiagen, Venlo, Netherlands) and evaluated for size distribution using the Agilent 4150 TapeStation with HS D1000 ScreenTape (Agilent, Santa Clara, CA, United States). Sequencing was conducted with single end reads (100 bp) at a depth exceeding 10 million reads per sample. miRNA expression levels were quantified using the Qiagen GeneGlobe platform (Qiagen) with UMI-based correction.

### Primary cultured cortical neurons

Pregnant female Sprague-Dawley (SD) rats were supplied by BioLASCO Taiwan Co., Ltd. (Yilan, Taiwan). All animals (one rat/individually ventilated cage) were housed in an air-conditioned room (22 ± 2°C) on a 12 hr-light/dark cycle (07:00–19:00 h light) and had free access to food and water. To prepare primary cultured cortical neurons, embryonic day 17 fetal rat brains were obtained from pregnant female SD rats of 17-day gestation which were sacrificed by an overdose of Zoletil^®^ (Virbac, Taipei, Taiwan) to minimize pain or discomfort used. The use of animals and all experiments conducted were under approved protocols from the Institutional Animal Care and Use Committee (IACUC) of Taipei Veterans General Hospital, Taipei, Taiwan. The approval number is IACUC2022-235. In addition, the authors complied with the ARRIVE guidelines. All experiments were performed in accordance with relevant guidelines and regulations.

Cerebral cortices of fetal rats were isolated and dissociated mechanically. The dissociated cells were suspended in the Basal Medium Eagle medium (BME, Thermo Fisher Scientific) containing 20% fetal bovine serum, and were seeded onto a 35-mm culture dish (IWAKI, Tokyo, Japan) with a density of 5 × 10^6^ cells per dish. Afterwards, cells were maintained with serum-free NB medium supplemented with B27 (Thermo Fisher Scientific) in the incubator with 5% CO_2_ at 37°C. Hemin-induced neurotoxicity was established by exposing primary cultured cortical neurons with hemin (30 μM).

### Cytotoxicity assay

A modified sulforhodamine B (SRB) assay was employed to measure cell viability. At the end of experiment, cells in 96-well plates were washed with phosphate-buffered saline (PBS) and 10% trichloroacetic acid (TCA, Merck, Boston, MA, United States) and were then incubated at 4°C for 1h. Afterwards, TCA solution was removed, and the cells were washed with double distilled H_2_O. Cells were then incubated with SRB solution (0.4% in 1% acetic acid, Merck) for 10 min. Afterwards, SRB solution was removed, and the cells were washed with 1% acetic acid. After removing acetic acid, the sample was air-dried and 20 mM unbuffered Tris base was used to dissolve the resulting formazan product. The absorption was measured by an ELISA reader (TECAN Sunrise, Männedorf, United States) at 540 nm with a reference wavelength of 690 nm.

### Western blots analysis

At the end of 16-h treatments, the cells were collected, washed with phosphate buffered saline (PBS), and lysed in radioimmunoprecipitation assay (RIPA, Cell Signaling Tech. Beverly, MA, United States) lysis buffer containing 20 mM Tris HCl, 150 mM NaCl, 1% (v/v) NP-40, 1% (w/v) sodium deoxycholate, 1 mM ethylenediaminetetraacetates (EDTA), 0.1% (w/v) sodium dodecyl sulfate polyacrylamide (SDS) and 0.01% (w/v) sodium azide (pH 7.5) for 20 min on ice. Lysates were then centrifuged at 13800 × *g* for 10 min, and the protein concentrations of supernatants were determined by Pierce BCA Protein Assay Kit (Thermo Fisher Scientific). Protein samples (30 μg) were run on 12–13.5% SDS-polyacrylamide gel electrophoresis and then transferred onto a polyvinylidene difluoride (PVDF, Bio-Rad, Hercules, CA, United States) at 100 V for 120 min. Blots were probed with primary antibodies including antibodies against GAP43 (Cell Signaling Technology), iNOS, COX-2, HO-1 (StressGen, Victoria, CA, United States), GPX4 and active-caspase 3 (Cell Signaling Technology) overnight at 4°C. After incubation of primary antibodies, the membrane was washed and incubated with a secondary antibody for 1 h at room temperature. The secondary antibodies were horseradish peroxidase-conjugated secondary IgG (Chemicon, Temecula, CA, United States). The immunoreaction was visualized using Amersham Enhanced Chemiluminescence (Amersham Pharmacia Biotech, Piscataway, NJ, United States). After this measurement, the bound primary and secondary antibodies were stripped by incubating the membrane in stripping buffer (100 mM 2-mercaptoethanol, 2% SDS) at 50°C for 5 min. The membrane was reprobed with a primary antibody against β-actin (Millipore, Billerica, MA, United States).

### Immunofluorescent staining

At the end of treatments, the primary cultured cortical neurons were fixed with 4% paraformaldehyde (Merck). Cells were then washed with 0.1 M PBS, incubated with 0.3% Triton X-100 (Sigma) and 1% goat serum (GS; Jackson ImmunoResearch, West Grove, PA, United States), and blocked with 3% GS for 60 min. Next, cells were processed for immunostaining using monoclonal antibody specific for rat Neu N (Cell Signaling Technology) in 1% GS-PBS at 4°C for 24 h. The cells were then incubated in fluorescein conjugated-IgG (FITC) (Jackson ImmunoResearch) for 1 h at room temperature, mounted in glycerol (Merck) and exosome stained with PKH26 (Sigma). Controls consisted of omissions of primary antibodies. The sections were visualized by a fluorescence confocal microscope (Olympus FluoView, Norfolk, VA, United States).

### Statistics

All data are expressed as the mean ± S.E.M. The results were analyzed by one-way analysis of variance (one-way ANOVA) followed by the LSD test (cell viability and Western blot assay) or Tukey multiple comparison (NTA) as *post hoc* methods. The significance level was set at p < 0.05.

## Results

### Characterization of exosomes of hypoxic preconditioned CTX-TNA2 cells

**FIGURE 1:**
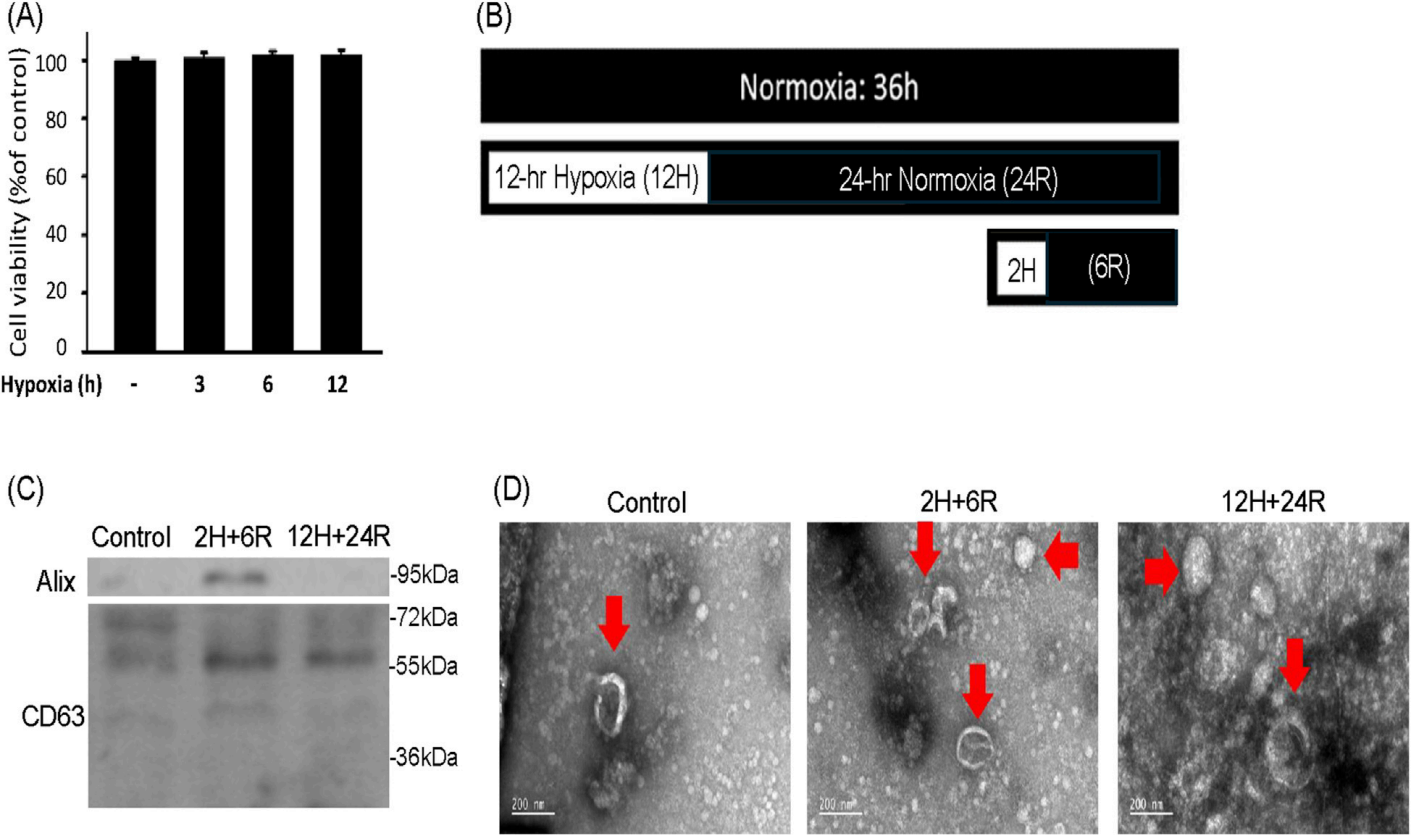
Characterization of exosomes secreted from CTX-TNA2 cells subjected to different hypoxic preconditionings. **(A)** CTX-TNA2 cells were treated with hypoxia for 3, 6, and 12 h. Cell viability was measured using SRB assay. Values are the mean ± S.E.M. (n = 3/each group). No statistical signifijcance was observed using one-way ANOVA followed by the LSD test as *post hoc* method. **(B)** The diagram illustrated two different hypoxic preconditioings of CTX-TNA2 cells for exosomes collection. One was 2H/6R exosomes which were obtained from CTX-TNA2 cells exposed to 2-h hypoxia followed by 6-h normoxia. The other was 12H/24R exosomes which were obtained from CTX-TNA2 cells exposed to 12-h hypoxia followed by 24-h normoxia **(C,D)** 2H/6R exosomes, 12H/24R exosomes and control exosomes obtained from 1 × 10^6^ CTX-TNA2 cells were used. Western blot assay was employed to measure exosomal markers, including Alix and CD63 **(C)**. TEM images of control exosomes, 2H/6R exosomes and 12H/24R exosomes were performed **(D)**. Representative exosomes were indicated by arrows in each images. Scale bar = 200 nm.

To delineate the effect of hypoxic preconditionings on exosome secretion, CTX-TNA2 astrocyte cells were treated with 3, 6, and 12 h hypoxia. SRB assay showed that hypoxia (1% oxygen) for 3, 6, 12 h did not cause significant cell loss of CTX-TNA2 cells (Figure 1A). Therefore, 2-h hypoxia followed by 6-h normoxia (2H/6R) and 12-h hypoxia followed by 24-h normoxia (12H/24R) were designed to treat CTX-TNA2 cells to harvest exosomes (Figure 1B). Western blot assay showed that compared with the control, Aix and CD 63 levels were elevated in the 2H/6R exosomes and 12H/24R exosomes (Figure 1C). TEM analysis showed that control exosomes, 2H/6R exosomes and 12H/24R exosomes all exhibited a spherical morphology with an intact lipid bilayer membrane (Figure 1D). Furthermore, the NTA assay showed that the sizes of 2H/6R exosomes and 12H/24R exosomes were slightly larger but not statistically different from the control exosomes, ranging from 89.8 ± 9.9 nm in the control exosomes to 99.4 ± 12.7 mm in 2H/6R exosomes and 105.1 ± 22.3 nm in the 12H/24R exosomes (n = 3/group) (Table 1). At the same time, the concentrations of exosomes particles were not statistically different among 3 exosomes (Table 1). Furthermore, no statistical differences were observed in zeta potentials of 3 exosomes (Table 1).

**TABLE 1:** NTA assay of 2H/6R exosomes and 12H/24R exosomes. 2H/6R exosomes were obtained from CTX-TNA2 cells exposed to 2-h hypoxia followed by 6-h normoxia. The other was 12H/24R exosomes obtained from cells exposed to 12-h hypoxia followed by 24-h normoxia. NTA assay and zeta potential assay were performed. Values are the mean ± S.E.M. (n = 3/each group). No statistical signifijcance was observed using one-way ANOVA followed by the Tukey multiple comparison as *post hoc* method.

Exosomes’ conditions	Size (nm)	Concentration (particles/mL)	Zeta potential (mV)
Control exosomes	89.8 ± 9.9	1.25 ± 0.35 × 10^10^	−8.57 ± 0.27
2H/6R exosomes	99.4 ± 9.0	1.04 ± 0.29 × 10^10^	−8.72 ± 0.87
12H/24R exosomes	105.1 ± 22.3	1.40 ± 0.64 × 10^10^	−7.48 ± 0.80

### Differential effects of exosomes of hypoxic preconditioned CTX-TNA2 cells on hemin-induced cell death and morphological changes

**FIGURE 2:**
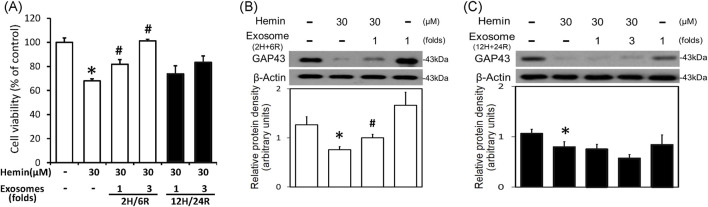
Differential effects of 2H/6R exosomes and 12H/24R exosomes on hemin neurotoxicity in primary cultured cortical neurons. **(A)** Primary cultured cortical neurons were treated with hemin (30 μM) plus 2H/6R exosomes obtained from 1 × 10^6^ CTX-TNA2 cells (as 1-fold) and 12H/24R exosomes (1 fold, 3 folds) for 16 h. Cell death was measured by SRB assay. **(B,C)** Primary cultured cortical neurons were treated with hemin (30 μM) plus 2H/6R exosomes (1-fold) **(B)** and 12H/24R exosomes (1 fold and 3 folds) for 16 h. Western blot assay was employed to measure GAP43. Each lane contained 30 μg protein for all experiments. Graphs show statistic results from relative optical density of bands on the blots. Values are the mean ± S.E.M. (n = 3/each group). *, p < 0.05 statistically significant in the hemin groups compared with the control groups; #, P < 0.05 in hemin plus exosomes compared with hemin alone by one-way ANOVA followed by the LSD test as *post hoc* method.

**FIGURE 3:**
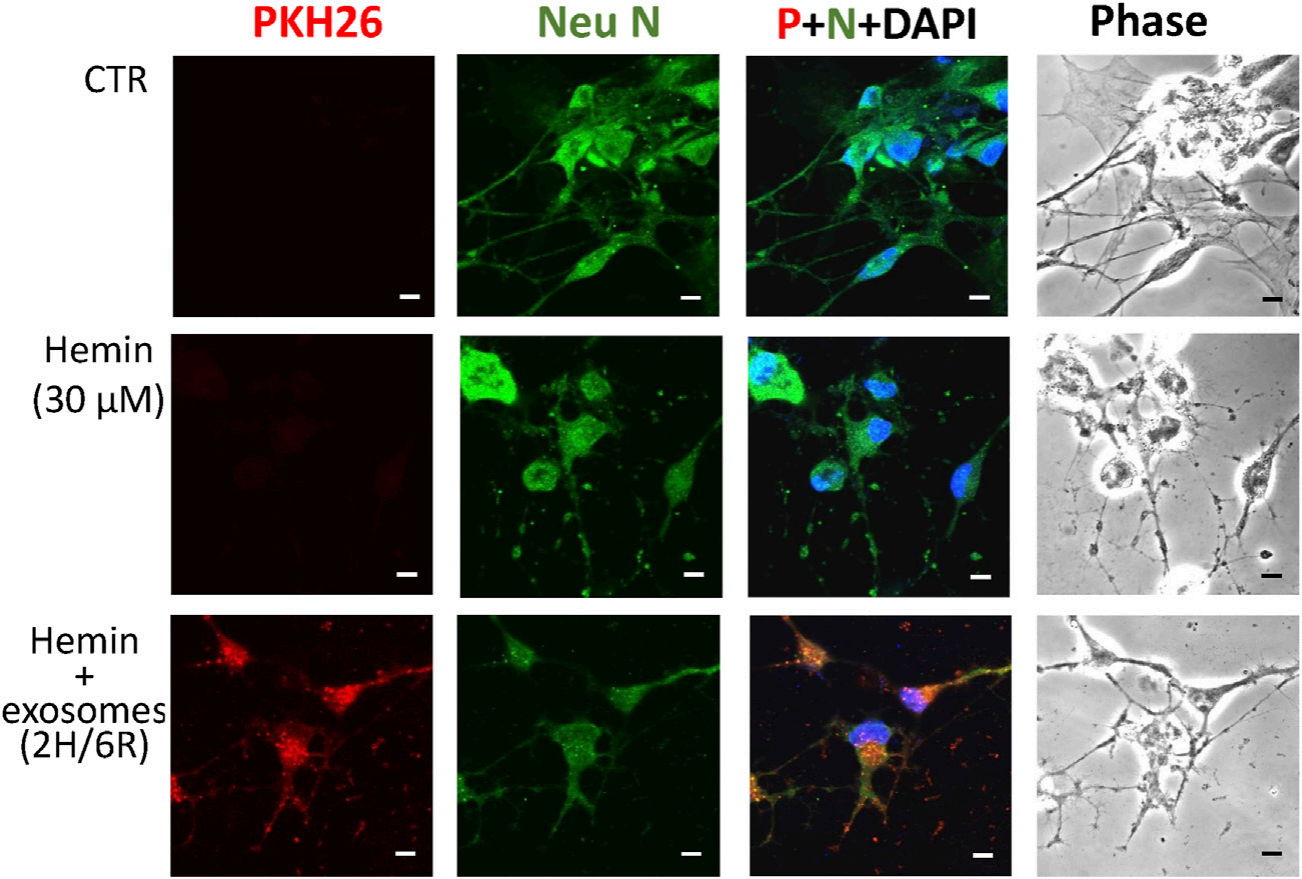
Differential effects of 2H/6R exosomes and 12H/24R exosomes on hemin-induced impairment of neurite outgrowth in primary cultured cortical neurons. Primary cultured cortical neurons were treated with hemin (30 μM) plus 2H/6R exosomes obtained from 1 × 10^6^ CTX-TNA2 cells for 1 h. The cells were immunostained with Neu N, PHK26 and DAPI. Calibration: 10 μm. The results were duplicated.

To delineate the effects of 2H/6R exosomes and 12H/24R exosomes, a hemin-induced neurotoxicity model was established in primary cultured cortical neurons. The SRB assay showed that 16-h incubation of hemin (30 μM) increased neuronal death (Figure 2A). Co-incubation with 2H/6R exosomes concentration-dependently attenuated hemin-induced neuronal death. In contrast, 12H/24R exosomes did not significantly alter hemin-induced neuronal death (Figure 2A). At the same time, Western blot assay showed that hemin reduced GAP43 levels (a biomarker of neurite outgrowth). Co-incubation of 2H/6R exosomes, but not 12H/24R exosomes, attenuated hemin-induced reduction in GAP 43 levels, indicating that 2H/6R exosomes may attenuate hemin-induced reduction of neurite outgrowth (Figures 2B,C). Moreover, the immunofluorescent staining data showed focal bead-like swellings, neuritic beading and discontinuities of neurites in hemin-treated primary cultured cortical neurons after 1-h incubation of hemin, indicating hemin-induced impairments in neurite outgrowth (Figure 3). Co-incubation of 2H/6R exosomes significantly attenuated hemin-induced neurite impairment (Figure 3). At the same time, intensive PKH26 fluorescence (a fluorescent dye specific for exosomes) was detected in the cytosol of primary cultured cortical neurons treated with hemin plus 2H/6R exosomes, suggesting that exosomes were successfully endocytosed by primary cultured cortical neurons.

### Differential effects of exosomes of hypoxic preconditioned CTX-TNA2 cells on hemin-induced neuroinflammation, HO-1 expression and programmed cell death

**FIGURE 4:**
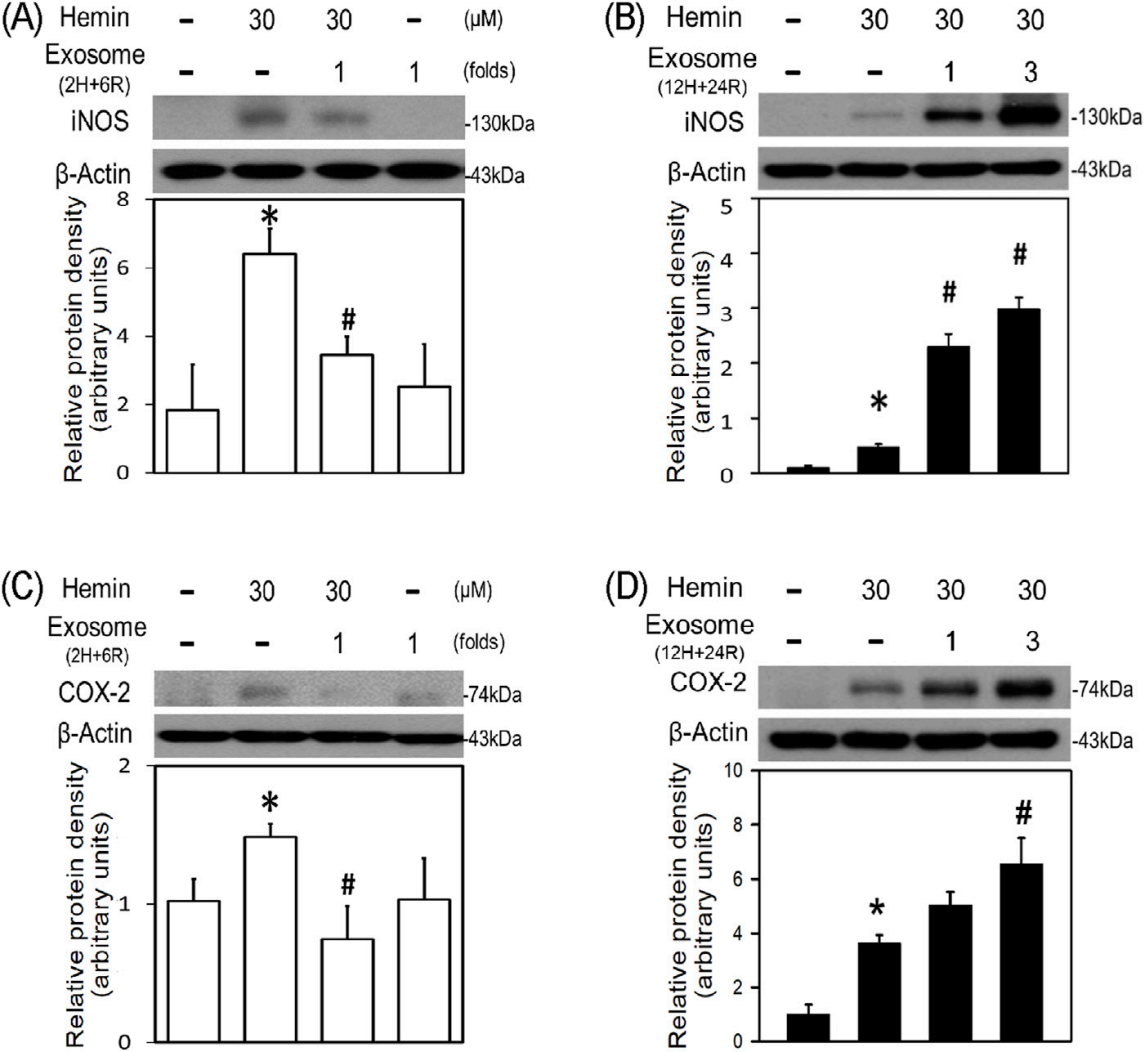
Differential effects of 2H/6R exosomes and 12H/24R exosomes on hemin-induced neuroinflammation in primary cultured cortical neurons. **(A,C)** Primary cultured cortical neurons were treated with hemin (30 μM) plus 2H/6R exosomes obtained from 1 × 10^6^ CTX-TNA2 cells (as 1 fold) for 16 h **(B,D)** Primary cultured cortical neurons were treated with hemin (30 μM) plus 12H/24R exosomes (1 fold and 3 folds) for 16 h. Western blot assay was employed to measure iNOS **(A,B)** and COX-2 **(C,D)**. Each lane contained 30 μg protein for all experiments. Graphs show statistic results from relative optical density of bands on the blots. Values are the mean ± S.E.M. (n = 3/each group). *, p < 0.05 statistically significant in the hemin groups compared with the control groups; #, P < 0.05 in hemin plus exosomes compared with hemin alone by one-way ANOVA followed by the LSD test as *post hoc* method.

**FIGURE 5:**
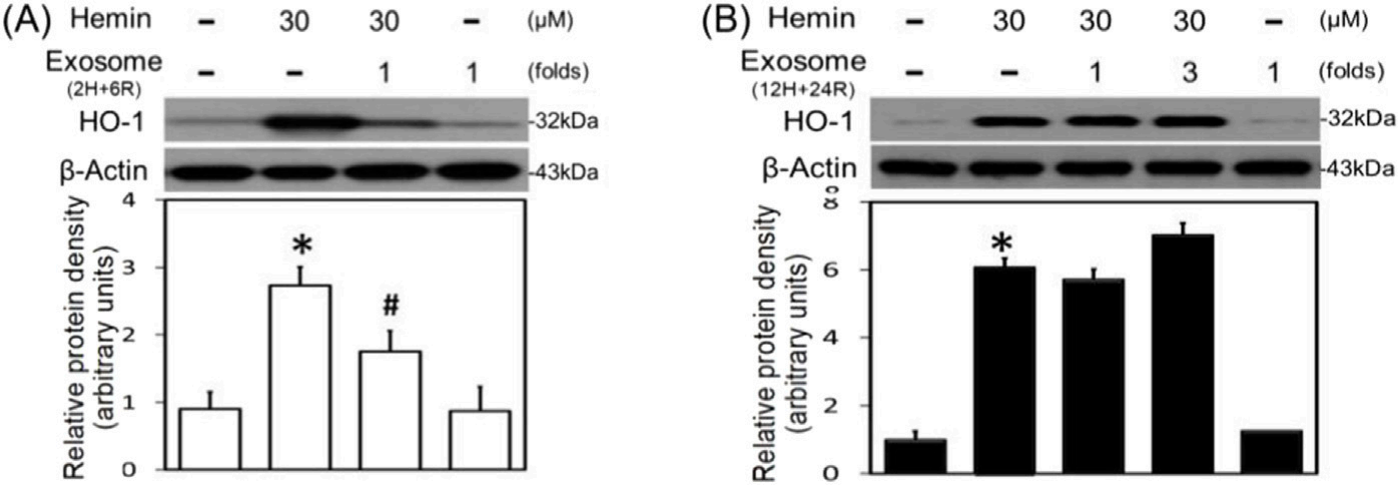
Differential effects of 2H/6R exosomes and 12H/24R exosomes on hemin-induced HO-1 expression in primary cultured cortical neurons. **(A)** Primary cultured cortical neurons were treated with hemin (30 μM) plus 2H/6R exosomes obtained from 1 × 10^6^ CTX-TNA2 cells (as 1-fold) for 16 h. **(B)** Primary cultured cortical neurons were treated with hemin (30 μM) plus 12H/24R exosomes (1 fold and 3 folds) for 16 h. Western blot assay was employed to measure HO-1. Each lane contained 30 μg protein for all experiments. Graphs show statistic results from relative optical density of bands on the blots. Values are the mean ± S.E.M. (n = 3/each group). *, p < 0.05 statistically significant in the hemin groups compared with the control groups; #, P < 0.05 in hemin plus exosomes compared with hemin alone by one-way ANOVA followed by the LSD test as *post hoc* method.

To further investigate the differential effects of 2H/6R exosomes and 12H/24R exosomes, iNOS and COX-2 (two proinflammatory biomarkers) were measured in the primary cultured cortical neurons. Western blot assay demonstrated hemin (30 μM)-induced neuroinflammation by increasing iNOS (Figures 4A,B) and COX-2 (Figures 4C,D) in hemin-treated primary cultured cortical neurons. Co-incubation with 2H/6R exosomes significantly prevented hemin-induced elevations in iNOS (Figure 4A) and COX-2 (Figure 4C). In contrast, 12H/24R exosomes further increased hemin-elevated iNOS (Figure 4B) and COX-2 (Figure 4D). These data indicated opposite effects of 2H/6R exosomes and 12H/24R exosomes on hemin-induced neuroinflammation. At the same time, we investigated the effects of exosomes on HO-1 expression (an enzyme catalyzing hemin). Western blot assay showed that co-incubation of H/6R exosomes but not 12H/24R significantly attenuated hemin-induced elevation in HO-1 expression (Figure 5), suggesting that 2H/6R exosomes can reduce hemin-induced HO-1 expression.

**FIGURE 6:**
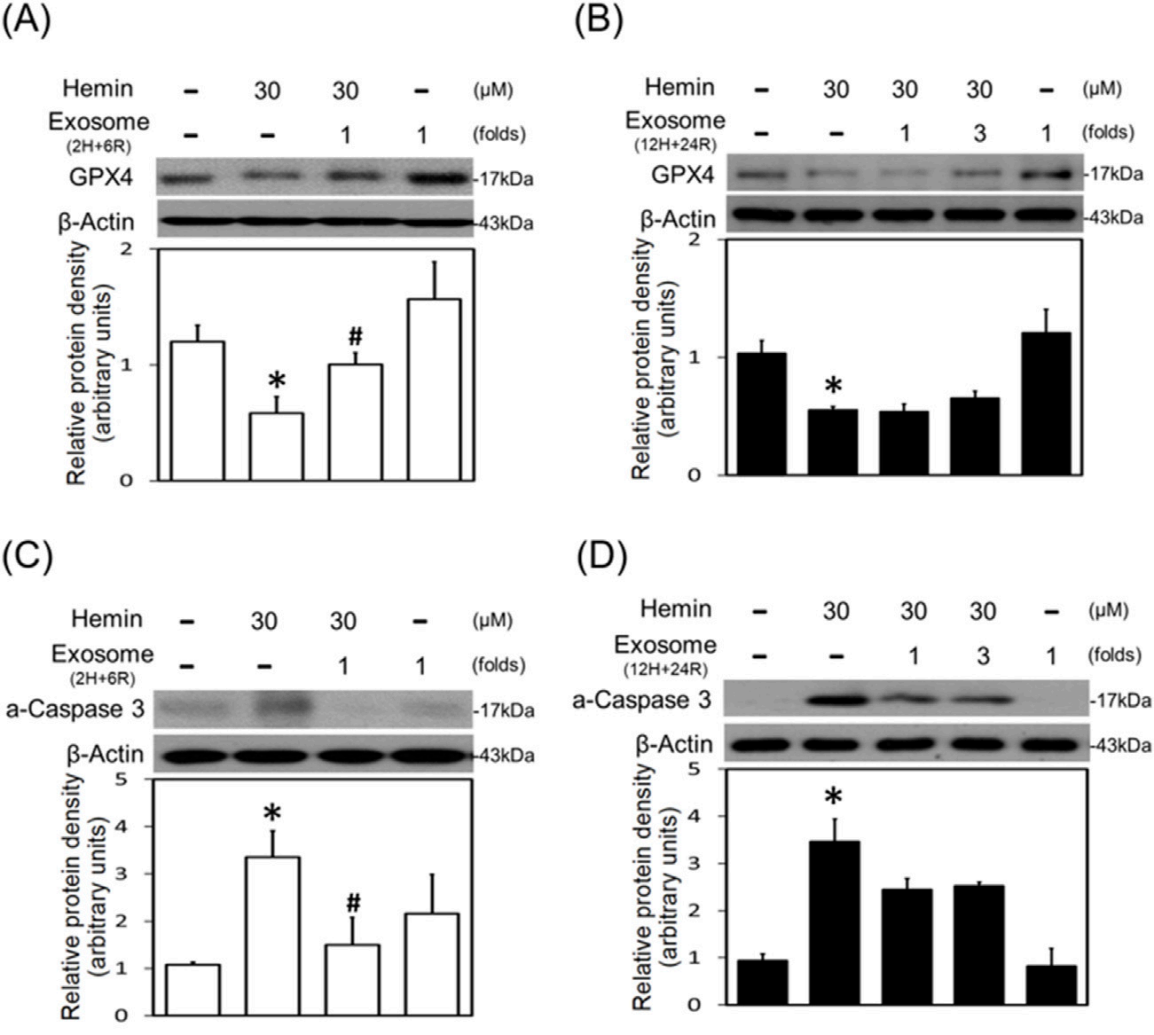
Differential effects of 2H/6R exosomes and 12H/24R exosomes on hemin-induced programmed cell death in primary cultured cortical neurons. **(A,C)** Primary cultured cortical neurons were treated with hemin (30 μM) plus 2H/6R exosomes obtained from 1 × 10^6^ CTX-TNA2 cells (as 1 fold) for 16 h **(B,D)** Primary cultured cortical neurons were treated with hemin (30 μM) plus 12H/24R exosomes (1 fold and 3 folds) for 16 h. Western blot assay was employed to measure GPX4 **(A,B)** and active-caspase 3 **(C,D)**. Each lane contained 30 μg protein for all experiments. Graphs show statistic results from relative optical density of bands on the blots. Values are the mean ± S.E.M. (n = 3/each group). *, p < 0.05 statistically significant in the hemin groups compared with the control groups; #, P < 0.05 in hemin plus exosomes compared with hemin alone by one-way ANOVA followed by the LSD test as *post hoc* method.

The neurotoxic mechanisms underlying hemin-induced neuronal death were investigated by measuring glutathione hydroperoxidase 4 (GPX4, a biomarker of ferroptosis) and active caspase 3 (a biomarker of apoptosis). Western blot assay demonstrated that hemin (30 μM) reduced GPX4 (Figures 6A,B) and increased active caspase 3 (Figures 6C,D). Co-incubation with 2H/6R exosomes attenuated hemin-induced reduction in GPX4 (Figure 6A) and elevation in active caspase 3 (Figure 6C). In contrast, 12H/24R exosomes did not alter hemin-induced reduction in GPX4 (Figure 6B) and elevation in active caspase 3 (Figure 6D), indicating indicate that 2H/6R exosomes appear to reduce hemin-induced ferroptosis and apoptosis.

### Diverse cargos in 2H/6R exosomes and 12H/24R exosomes

**FIGURE 7:**
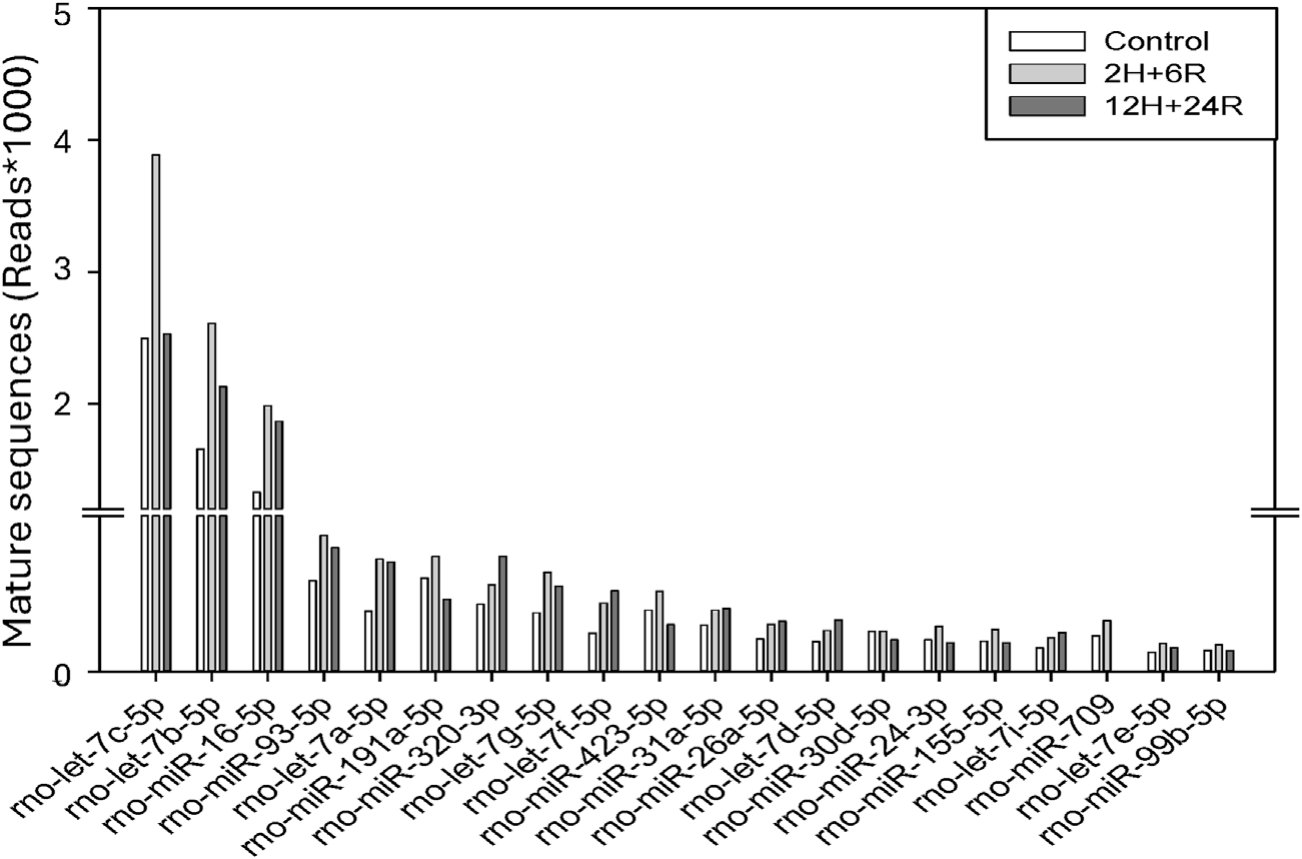
Cargos in control exosomes, 2H/6R and 12H/24R exosomes by miRNA sequencing analysis. Sequencing was performed with single-end reads (100 bp) at a depth exceeding 10 million reads per sample. miRNA expression levels were analyzed on the Qiagen GeneGlobe platform with UMI-based correction.

So far, we have demonstrated differential effects of 2H/6R exosomes and 12H/24R exosomes on hemin-induced neurotoxicity; 2H/6R exosomes, but not 12H/24R exosomes, possessed a neuroprotective activity. The cargos, especially miRNA in both exosomes were analyzed using RNA Sequencing Analysis. We found significant changes in the miRNA expression profiles, including the expression levels of let-7c-5p, miR-191a-5p, and miR-709 in 2H/6R exosomes (Figure 7).

## Discussion

In the present study, the effects of hypoxia on exosomes obtained from CTX-TNA2 cells exposed to different hypoxic preconditionings were delineated using hemin-induced neurotoxicity as follows. First, compared to the control exosomes, 2H/6R exosomes and 12H/24R exosomes did not show significant difference in exosomes secretion. Secondly, 2H/6R exosomes showed neuroprotective properties by attenuating hemin-induced neurotoxicity. However, 12H/24R exosomes appear to be proinflammatory by augmenting hemin-induced neuroinflammation. Our study showed that exosomes from CTX-TNA2 cells subjected to different hypoxic preconditionings possess opposite properties, suggesting that hypoxia plays a double-edged role in exosome’s functions.

Hypoxia, a condition of insufficient oxygen, is reportedly an external factor that stimulates exosome secretion [22, 23]. Several *in vitro* studies on cancer biology have demonstrated that incubation of cancer cells under hypoxia (1% oxygen for 24–72 h) which mimicked hypoxia in the tumor microenvironment, increased exosome releases [14, 23]. At the same time, exosomes from hypoxic cancer cells contain proangiogenic miRNA and growth factors for angiogenesis and cancer cell proliferation, respectively [14, 24], indicating that in addition to exosome secretion, hypoxia is capable of altering exosome’s properties [23, 24]. To support this notion, two different hypoxic preconditionings were designed to treat CTX-TNA2 cells and collect exosomes from the cultured medium individually. Our data showed that hypoxia preconditionings significantly increased exosome biomarkers and slight increases in exosome size. Due to the lack of differences in particle concentration, one possible explanation is that more exosome biomarkers are expressed in each exosome. Our data suggested that hypoxic preconditioning did not significantly affect exosome secretion.

In contrast, hypoxic preconditioning significantly changed the properties of exosomes. To delineate the exosome’s properties, we employed hemin [18] which is commonly used to mimic ICH-related secondary injury [25], including cytotoxicity, neurite impairment, HO-1 expression, neuroinflammation and program cell death. Hemin, a byproduct of hemoglobin degradation [26], is known as an HO-1 inducer to catalyze the degradation of heme/hemin and release iron [27], a Fenton’s reagent which induce oxidative injury and subsequent cell death [28]. We found that 2H/6R exosomes attenuated hemin-induced neuronal death, neurite impairment and neuroinflammation. More importantly, 2H/6R exosomes significantly inhibited hemin-elevated HO-1 levels which resulted in less iron accumulation and ameliorated subsequent apoptosis and ferroptosis, a programmed cell death related to iron metabolism. In contrast to the 2H/6R exosomes-induced neuroprotection, 12H/24R exosomes significantly augmented hemin-induced elevation in iNOS and COX-2, indicating that 12H/24R exosomes is proinflammatory. Due to the opposite effects of 2H/6R exosomes and 12H/24R exosomes on hemin-induced neurotoxicity, our data further support that hypoxia plays a double-edged role in regulating exosome functions.

A significant body of studies has focused on the pathological effects of hypoxia on CNS, including ICH. The pathophysiology of hypoxia depends on the duration of hypoxia, including acute, subacute and chronic hypoxia [29]. In addition to 2H/6R and 12H/24R, more hypoxia-renormoxia treatments were tested, including 3H/24R, 6H/24R, and 12H/24R (unpublished data). To avoid obtaining exosomes from unhealthy cells, we chose hypoxic duration from 2 to 12 h because hypoxia duration less than 12 h did not cause significant cytotoxicity. All cells are capable of releasing exosomes [1–3], including brain cells. In addition to CTX-TNA2 cells, we have tested different hypoxia-renormoxia conditions on primary cultured cortical neurons. The exosomes from primary cultured cortical neurons showed different biological activities from those from CTX-TNA2 (unpublished data). Accordingly, types and conditions of donor cells are the critical factors for exosome production [30].

To obtain “good” exosomes from hypoxic cells, it is important to design an ideal hypoxic condition, including optimal duration (2–72 h) [14] and intensity (0.1%–5% oxygen) [13, 31]. For example, exosomes obtained from MSCs subjected to 48-h hypoxia (1% O2) were reportedly more efficacious to bone fracture healing [10]. In addition, exosomes from hypoxic donor cells (3% for 24 h) were found to be neuroprotective to attenuate program cell death induced by OGD/reperfusion [32]. “As an important intercellular mediator, “cargos” in the exosomes, including miRNAs, are the key element for its functions [30]. For example, miR-92b-3p in the exosomes released from OGD-treated astrocytes has been suggested to mediate the neuroprotection against OGD-induced neurotoxicity [33]. Furthermore, exosome-delivered miR216a-5p from hypoxic preconditioned-MSCs is suggested to repair spinal cord injury [34]. In the present study, we identified several miRNAs elevated in the 2H/6R exosomes, including let-7c-5p which has been suggested to be neuroprotective against cerebral ischemia [35]. Accordingly, 2H/6R treatment may produce “good” exosomes for neuroprotection.

In conclusion, hypoxia has been used as a strategy to enhance exosome secretion. However, beyond the quantity of released exosomes, it is equally important to characterize how hypoxia simultaneously affects exosomes’ properties. Given that function of exosomes is closely tied to the conditions of donor cells, our data demonstrated that CTX-TNA2 cells exposed to varying hypoxia-renormoxia treatments produce exosomes with opposite functional profiles. The present study demonstrates the complex effects of hypoxia as well as a double-edged role of hypoxia on exosome functionality.

## Data Availability

The original contributions presented in the study are included in the article/supplementary material, further inquiries can be directed to the corresponding author.
