# Corrigendum: A double-edged effect of hypoxia on astrocyte-derived exosome releases

**DOI:** 10.3389/ebm.2025.10735

**Published:** 2025-07-23

**Authors:** Yang Jie Tseng, Hui-Ju Huang, Chien-Hui Lin, Anya Maan-Yuh Lin

**Affiliations:** ^1^Ph.D. Program in Regulatory Science and Policy, National Yang-Ming Chiao-Tung University, Hsin-Chu, Taiwan; ^2^Department of Medical Research, Taipei Veterans General Hospital, Taipei, Taiwan; ^3^Institute of Physiology, National Yang-Ming Chiao-Tung University, Hsin-Chu, Taiwan; ^4^Department of Pharmacy, National Yang-Ming Chiao-Tung University, Hsin-Chu, Taiwan

**Keywords:** hypoxic preconditioning, double-edged role, exosomes, hemin, CTX-TNA2

In the original article, there was a mistake in Figure 6 as published. The error was due to the inadvertent insertion of an incorrect version of the figure during the final submission process. Specifically, the published figure mistakenly displayed HO-1 (A, B) and GPX4 (C, D), instead of the correct picture, which should show GPX4 (A, B) and active-caspase 3 (C, D), as measured by Western blot assay. The corrected Figure 6 appears below.

**FIGURE 6:**
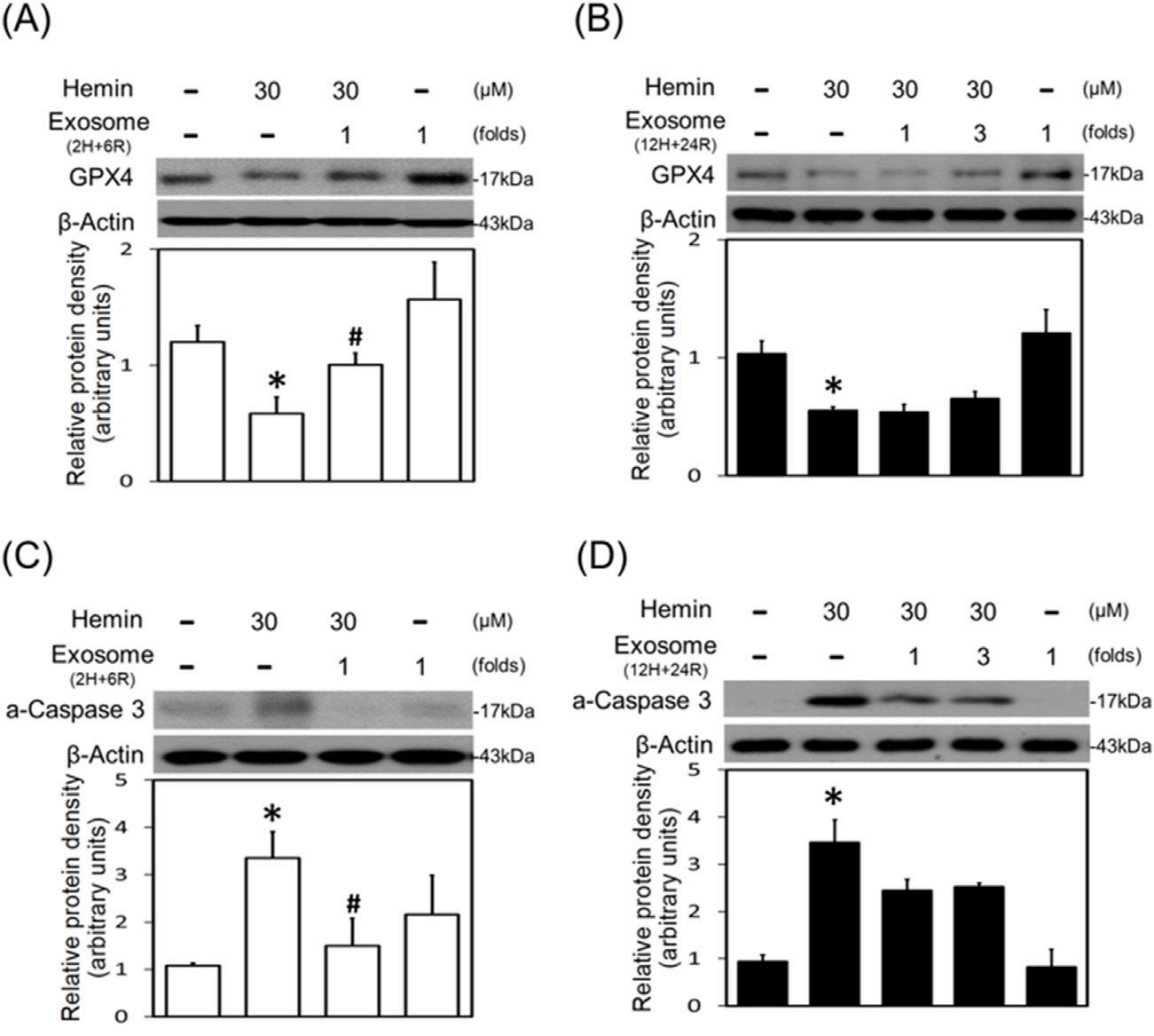
Differential effects of 2H/6R exosomes and 12H/24R exosomes on hemin-induced programmed cell death in primary cultured cortical neurons. **(A,C)** Primary cultured cortical neurons were treated with hemin (30 μM) plus 2H/6R exosomes obtained from 1 × 10^6^ CTX-TNA2 cells (as 1 fold) for 16 h **(B,D)** Primary cultured cortical neurons were treated with hemin (30 μM) plus 12H/24R exosomes (1 fold and 3 folds) for 16 h. Western blot assay was employed to measure GPX4 **(A,B)** and active-caspase 3 **(C,D)**. Each lane contained 30 μg protein for all experiments. Graphs show statistic results from relative optical density of bands on the blots. Values are the mean ± S.E.M. (n = 3/each group). *, p < 0.05 statistically significant in the hemin groups compared with the control groups; #, P < 0.05 in hemin plus exosomes compared with hemin alone by one-way ANOVA followed by the LSD test as *post hoc* method.

The authors apologize for this error and state that this does not change the scientific conclusions of the article in any way. The original article has been updated.

